# Lifestyles through Expenditures: A Case-Based Approach to Saving

**DOI:** 10.15195/v3.a28

**Published:** 2016-08-03

**Authors:** Lisa A. Keister, Richard Benton, James Moody

**Affiliations:** a Duke University; b University of Illinois

**Keywords:** saving, cluster analysis, lifestyles, spending, case-based social science

## Abstract

Treating people as cases that are proximate in a behavior space—representing lifestyles—rather than as markers of single variables has a long history in sociology. Yet, because it is difficult to find analytically tractable ways to implement this idea, this approach is rarely used. We take seriously the idea that people are whole packages, and we use household spending to identify groups who occupy similar positions in social space. Using detailed data on household consumption, we identify eight positions that are clearly similar in lifestyle. We then study how the lifestyles we identify are associated with saving, an important measure of household well-being. We find that households cluster into distinct lifestyles based on similarities and differences in consumption. These lifestyles are meaningfully related in social space and save in distinct ways that have important implications for understanding inequality and stratification.

Sociologists tend to agree that people are more than the variables that can be used to describe them, but efforts to study people as whole packages are surprisingly rare. Social theorists have described how people cluster based on their engagement with material culture (Simmel 1972) or position in wider relational fields (Bourdieu 1990). These approaches treat social variation as case-centered rather than variable-centered. In doing so, they take aspects of structural constraint, avocation, and preference as complete packages that capture social position. Sobel and Rossi (1981) referred to these whole packages as *lifestyles*, or the “set of observable behavioral choices that individuals make.” Building on Bourdieu’s conception (Bourdieu and Passeron 1977), Sobel and Rossi (1983) noted that the behaviors making up a lifestyle are constrained by “needs and resources, the totality of material culture, and the rules of the political economy that govern the distribution of cultural elements.” Similarly, in his critique of variable-centered causal analysis, Abbott (1998) called for more accurate, rich, descriptive typologies that better reflect people’s lives. The core idea in these approaches is that information from a set of related behaviors can identify groups of households that are similarly situated in social space, who face similar constraints and opportunities, and whose behaviors are similarly oriented as a result. There have been some notable, recent efforts to use a case-centered approach (Brint, Riddle, and Hanneman 2006; Garip and Asad 2014; Martin 2011), and this style of thinking is common in ethnographic work; however, this approach is still rare in large-scale quantitative sociology.

Much of the challenge in taking the case-centered approach seriously is finding analytically tractable ways to identify those with similar lifestyles. We propose that the way people spend money captures rich information about the behaviors, attitudes, and life conditions that interact to create lifestyles; thus, spending offers a unique opportunity to answer the call to move beyond variable-centered approaches to social life. Particular spending decisions—such as decisions to support a political or religious group, buy certain clothing, get a tattoo—when considered in the context of other spending decisions across and within households, locate households in a multi-dimensional social space. That is, how households allocate available income across spending options offers insight into their priorities, habits, and identities as well as the constraints within which they live; all of these would otherwise be difficult to measure and incomplete if viewed in isolation. Spending allocations also reflect important elements of structural position, class status, and geographic location because race and ethnicity, education, income, and related traits (e.g., area of residence) can affect both the availability and cost of goods and services and the way households allocate resources across possible expenditures. As a result, household spending across various categories reveals much about their lived experiences and how these translate into behaviors and actions. Bourdieu and Passeron (1977), Sobel and Rossi (1981), and others studying lifestyles in earlier sociology work (Featherstone 1987) recognized the potential of spending as a window into lifestyles, but this work fell out of favor at least partially because of data limitations at the time. Although household financial data are now readily available, spending decisions attract little attention in sociology beyond their use to understand cultural forms of consumption (DiMaggio and Useem 1978; Lamont and Molnár 2001).

In this article, we take seriously the notion of households as whole packages rather than simple markers of particular variables, and we examine how households cluster together based on spending allocations that define contemporary lifestyles. We then study how these lifestyles are associated with saving, an important indicator of household well-being that offers a rigorous case study of the idea that lifestyles matter. In what follows, we first elaborate on the motivations behind taking a case-centered approach, and we discuss some of the practical obstacles that have contributed to the limited use of this analytic strategy in prior social research. We then discuss the relevance of spending behavior for identifying cases or lifestyles, and we address the benefits of using lifestyle to understand saving behavior. We use data from the Consumer Expenditure Survey (CES), widely considered the most comprehensive and detailed data on household spending behavior, to study these ideas empirically. By moving beyond isolated marginal effects, our household-centered approach to lifestyle allows us to empirically identify clusters of emergent variation in survey data, identify household positions in social space (Bourdieu 1990), and capture some of the unique behaviors and practices associated with these positions that related to saving.

## From Variables to Lifestyles

The goal of the case-centered approach is to reorient analysis toward the complete package of behaviors associated with a particular social position and to use this information about social position to provide a more holistic explanation of behavior. While such work is comparatively rare in quantitative sociology, two broad variants of the approach appear in the literature. The objective of the first approach is the nearly complete endogenous reproduction of social position. Bourdieu’s notion of the *habitus* (Bourdieu 1977) and recent extensions of field and network theory (Martin 2003; Padgett and Powell 2012) typify this strategy. In such approaches, the set of behaviors centered on a particular domain defines a multidimensional space in which cases occupy positions defined by their relationship to all other positions in the space (Martin 2011). Thus the habitus is “the end product of structures which practices tend to reproduce in such a way that the individuals involved are bound to reproduce them, either by consciously reinventing or by subconsciously imitating already proven strategies as the accepted, most respectable, or even simplest course to follow. [They] … come to be seen as inherent in the nature of things” (Bourdieu 1976). This strong position effectively folds all variation into occupancy of the position and largely erases notions of general forces driving behavior.

The second strategy is a more pragmatic but less encompassing approach. This strategy merges a positional approach—typically focusing on specific topic domains—with more commonly used variable approaches to capture well-understood general variation in outcomes (Amato, Kane, and James 2011; Brint et al. 2006; Garip and Asad 2014; Kalleberg and Moody 1994). Such work often uses cluster analysis and similar analytic techniques to identify groups of people or households who share common ways of acting, but which might not otherwise be considered together. This approach allows an analyst to characterize position in the relational space through similarities and differences across multiple behaviors. An empirical identification of lifestyles would ideally be general enough to reproduce a considerable amount about the social space occupied by a case—consistent with the first strategy—but be sufficiently grounded that available data allow differentiation of respondents into particular groups while making use of well-known associations that guide behavior from variable-centered work—consistent with the second strategy.

The conceptual insight underlying these strategies is familiar to social scientists as interaction effects, in which the meaning of one variable is conditional on the meaning of another. For example, we know that African Americans living in majority white neighborhoods accumulate savings differently than those living in majority black neighborhoods because housing values vary and appreciate differently across these neighborhoods (Shapiro 2004). The logical but methodologically cumbersome extension of the interaction-effect strategy is to allow all variables to interact simultaneously, which is equivalent to asking how occupants of any point in an n-dimensional space defined by the variables behave. This approach to empirical understanding is the foundation of good ethnography: rather than identifying how outcomes vary by a single dimension, the ethnographer observes deeply contextualized behavior and identifies unique insights into how situations interact with numerous traits to shape behavior. Although cluster-analytic and similar approaches cannot reach the level of rich detail attained by good ethnography, the goal is to take seriously the idea that contextualized effects are better representations of life situations than single variables while retaining the powerful generalizability of large-scale surveys (Abbott 1998).

The role of social mechanisms in the case-centered approach has been debated. Garip (2014) suggests that cluster-based techniques allow us to identify mechanisms more precisely because they capture the interactive nature of variables. This contrasts strongly with work in causal modeling that focuses on the identification and complete isolation of effects of single variables (Morgan and Winship 2007) to best approximate a natural experiment. By using clusters within a regression context to adjust for potential demographic confounders, we are staking out a compromise position in this debate: we recognize that some features (age, employment status, education) have significant direct effects on social behavior, but we are deliberately open to discovering new associations characterized by lifestyle. The logical difference here is partially confounded empirically because clusters are induced observationally and the defining nature of a cluster is thus only observable indirectly based on distinctive cluster characteristics; a problem familiar to Bourdieu scholars as the difference between real classes and classes-on-paper (Bourdieu 1999). Thus, we engage in a partially post-hoc evaluation of the distinctive nature of identified clusters to help infer likely social mechanisms.

## Defining Lifestyles through Spending

Because American culture encourages and rewards consumption of both goods and services starting in early childhood, spending simultaneously reflects structural position, preferences, and identity. As such, expenditure profiles, or the way a household allocates available income across a large number of spending options, are excellent summary measures of the various incentives and pressures facing a household, including preferences as well as structural constraints and opportunities. Expenditure profiles are an ideal way to identify lifestyles because they span a wide set of socially relevant behaviors and provide clear indicators of household position in social space while remaining empirically tractable.

Expenditures are markers of social and economic structure because they reflect differences in income, education, family structure, area of residence, race/ethnicity, and institutionalized discrimination. Such factors affect expenditures in different ways but, more importantly, interact in nuanced ways to determine spending. For example, income necessarily constrains total spending, but it also dictates the allocation of spending across various categories: if rent, food, utilities, or other necessary expenses use a large proportion of income, a household may have little left for discretionary spending on luxuries such as movies, vacations, and other entertainment. Although unmeasured, living in a community of others who face similar constraints also likely changes one’s understanding of spending, reinforcing an implicit shared understanding of social position that is grounded in economic practice.

In the case of spending, individual and household traits and structural position mediate the association between financial lifestyle and inequality. Yet, income, education, race/ethnicity, family structure, area of residence, and other household traits are not randomly distributed in the population; rather, these markers vary together, and it is likely that expenditures partially reflect these traits. For instance, black–white differences in income and education are at least partially responsible for pronounced residential segregation in the United States (Iceland and Wilkes 2006; Massey and Denton 1993; Parisi, Lichter, and Taquino 2011), and residential segregation often results in low-income African Americans living in food/retail deserts and paying more for low-quality goods and services (Moore and Roux 2006; Myers et al. 2011). Thus, low-income African Americans, who already face hard budget constraints, may also endure higher costs for essential goods and services (Charles and Lundy 2013) that further constrain expenditure allocations. Added to this, some evidence suggests that African American and Latino consumers pay more for goods and services because of institutionalized discrimination, a pattern that is well known in housing redlining, but that also occurs in the sale of other goods and services (DiMaggio and Ostrower 1990; Feagin and Vera 1994; Feagin 1991).

Expenditures are also excellent indicators of preferences, habits, and identity. Decisions to support particular organizations financially, to spend discretionary income on entertainment and casual interests (e.g., collectors’ items, travel, dining out), or to purchase nonessential goods and services (e.g., designer clothing, spa treatments) reveals considerable information about preferences and habits. In addition, spending can be an important symbolic indicator; indeed, most sociological attention to spending behavior focuses on the symbolic meanings associated with consumption behavior and the implications of these actions for creating and maintaining group boundaries (Charles and Lundy 2013; Molnár and Lamont 2002; Warde and Martens 2000). This work builds on the notion that many purchases have both use value and symbolic value (Baudrillard 1998; Lamont and Molnár 2001); clothing, for example, is a necessary purchase, but choices about clothing types (e.g., colors, styles, designer/budget) can also be used to indicate distinctive social status or other elements of identity (Bennett et al. 2009; Bourdieu 1984; Warde 1997). Sociologists have extended these ideas to understand the unique role that a wide range of purchasing behaviors plays as semiotic tools in negotiating status in social and economic structures (Molnár and Lamont 2002; Warde 1997; Warde and Martens 2000). They show, for example, that African Americans have unique spending patterns that reflect nuanced negotiations of group boundaries and definitions (Lamont and Molnár 2001; Molnár and Lamont 2002) or conspicuous consumption as an indicator of success (Charles and Lundy 2013).

The logic here is akin to that of Bourdieu, in which habitus describes how structural position affects behaviors, lifestyles, and habits through internalized preferences, tastes, and cultural understandings (Bourdieu 1990). Because this activity includes social interaction and, in the case of spending, interdependent market forces, history and social organization conspire to make the distribution of the behavior space irregular or clumpy, allowing observers to identify similarities among cases by their proximate positions. This approach to identifying positions in the behavior space mirrors Bourdieu’s (1984:106) discussion of social classes as irreducible to a set of linear relationships, that is, as “the structure of relations between all the pertinent properties which gives its specific value to each of them and to the effects they exert on practices.” The valuation and meaning of these cultural practices are defined in relation to other practices in the field rather than emerging from some supposed essential qualities. Importantly, the combination of behaviors evident in a lifestyle captures social position in a way that cannot be identified by a single variable: for example, two households whose members listen to country music and hunt may still differ in significant ways if one owns a Prius and purchases food from a local co-op, whereas the other owns a Hummer and buys commodity food from Safeway. Thus, analyzing social position in the behavior space is likely to reveal patterns that would otherwise be overlooked or interpreted differently under a traditional variable-centric approach.

## Lifestyles and Saving

Household saving is a useful case study for understanding how lifestyles orient behaviors and, thus, might be associated with important outcomes. Saving and the assets that accumulate from saving are important components of financial well-being, yet Americans save very little. Accumulated savings can be used to pay for current needs and desires or retained to provide a buffer against unanticipated income interruptions, medical emergencies, accidents, natural disasters, and other crises. They can generate interest and dividends that can be consumed or reinvested—as in the purchase of real estate or financial assets—to create additional resources (Kennickell and Starr-McCluer 1997; Wolff 2010). Savings also improve educational attainment, occupational opportunities, political power, and social influence (Domhoff 2013; Freeland 2012). Perhaps most significantly, accumulated savings can be passed to future generations either as inter vivos transfers or inheritance to extend these benefits indefinitely (Avery and Rendall 2002; Gokhale and Villarreal 2006; Laitner 2001). Most Americans recognize the importance of saving, and awareness of asset inequality has also grown (Taylor et al. 2011), but saving rates in the United States remain surprisingly low. Between 2007 and 2012, Americans saved only 4 percent of their disposable incomes annually, with rates dropping to as low as 2 percent during the 2007–2009 recession (OECD Library 2013). By contrast, households in other developed countries such as Switzerland and Germany save as much as 14 percent of disposable income (OECD Library 2013).

A growing body of research acknowledges the importance of saving and accumulated assets; although surprising to non-specialists, this body of work shows significant variation in saving across all income and wealth levels. Indeed, the association between income and savings is moderate, with income accounting for only 25–36 percent of the variance in saved assets (Wolff 2010). Work explaining savings typically isolates one or two traits and studies how these variables correlate with saving. We know, for example, that saving is strongly associated with race and ethnicity (Avery and Rendall 2002; Campbell and Kaufman 2006; Oliver and Shapiro 2006; Shapiro 2004), education (Spilerman 2000), marriage and divorce (Zagorsky 2005), gender (Chang 2010), and religion (Keister and Sherkat 2013). Researchers have also paid some attention to the role of aggregate factors in predicting savings, including local barriers to accessing financial organizations, regional variation in market conditions, and, to a lesser extent, national-level business cycles and cohort processes (Kopczuk and Saez 2004; Shapiro 2004; Spilerman 2000; Wolff, Owens, and Burak 2011). This research provides powerful evidence isolating the association between saving behavior and key variables of interest; however, it focuses on particular variables as independent and disconnected influences while stripping the meaning of each variable from the context of other behaviors within which it is embedded. Instead of asking how households save, the focus is on average differences (holding all else constant) across households that vary on a single trait.

We propose that lifestyles better capture the relationship between a household’s social position and financial outcomes such as savings. There are at least three mechanisms through which lifestyles may be related to saving. The first is a relatively simple budget constraint, in which spending on particular items makes saving impossible. A second potential mechanism is knowledge based: embeddedness in a particular lifestyle might limit access to saving and investment options and knowledge about alternative financial instruments. Finally, a third mechanism is delay discounting, or the tendency to postpone current pleasure and needs for future rewards. These are likely overlapping mechanisms, as budget constraints create shortfalls that feed back into future crisis, whereas knowledge and access can create buffers that make future delay more palatable. For analytic clarity, we discuss these in turn below, but acknowledge they are likely overlapping.

To be clear, we are not simply arguing that households that spend more save less. There is little question that households who spend more necessarily save less. Rather, we are proposing that allocation differences across a wide array of spending categories provide unique insights into the lifestyle that defines a household, net of total spending. A focus on groups defined by spending behavior captures a facet of the interrelated processes and experiences that collectively generate household saving beyond what can be learned from the effects of individual variables. To avoid imposing our a priori notions about social groups on the data, we do not offer specific hypotheses about the lifestyle categories that are likely to emerge, nor do we anticipate how the emergent lifestyle categories will approach saving. Rather, our goal is to allow the data to guide our understanding of both the groups and their saving outcomes.

## Research Design

We use data from the 2011 Consumer Expenditure Survey (CES), a nationally representative survey of household expenditures. The Bureau of Labor Statistics (BLS) collects the CES data quarterly using a rotating panel design and typically obtains response rates between 70–75 percent; each sampled household is interviewed up to five times and completes an initial screening interview followed by interviews over four consecutive quarters. After the fifth interview, the household is replaced. The survey includes detailed household expenditure data as well as information on saving, work, income, and demographics. Each household in the sample we use was interviewed during 2011 and the first quarter of 2012, and each is interviewed up to five times. We use data on savings and financial traits from the fifth interview; that is, we limit our analysis to the fifth family interview and the expenditures that occurred in that quarter. Because the CES uses a rotating panel design and interviews are conducted throughout the year, the fifth interview does not occur in the same month for all households. Thus, we do not anticipate that seasonal consumption will systematically bias the results.

The CES includes information on more than 95 percent of total household expenditures (Bureau of Labor Statistics 2012), making it the single most comprehensive survey of household spending available and the best available data for our purposes. CES households are asked about their total expenditures across a wide variety of categories including food, entertainment, clothing, dwelling, and transportation. Debt payments (e.g., mortgage payments) are included as expenditures. The survey contains expenditure totals at the level of Universal Classification Codes (UCC), fine-grained product and service categories. The BLS aggregates the UCC expenditures into summary expenditure categories that indicate a household’s total expenditures during the three months prior to interview. Each of these summary expenditure categories is identified in Appendix A of the online supplement. Further detail on each summary expenditure category is available in the BLS public-use microdata documentation (Bureau of Labor Statistics 2012). These data are notably comprehensive: BLS routinely evaluates the accuracy and reliability of the survey instrument to ensure that expenditure data are as error-free as possible, and they conduct regular, extensive reviews to correct data errors and extreme values. BLS also uses multiple imputation to assign missing income data, although more than 99.39 percent of cases have complete income data prior to imputation. Of course, there are some downsides to using the CES: as with any survey data, the full extent of measurement error is unknown. In addition, the CES does not include as much detail for some variables, especially the saving variables, as some other data sets do. However, our comparisons of the saving and income measures with those available in other data sets (e.g., the National Longitudinal Survey, the Panel Survey of Income Dynamics, Current Population Survey) suggest that the CES measures are relatively accurate.

Spending is any purchase of goods and services that is essential (e.g., rent, mortgage, groceries), discretionary (e.g., entertainment, consumer electronics, vacations), or a combination of each (e.g., clothing, dining out). We consider these summary expenditure categories as a proportion of the household’s total expenditures (the sum of all expenditure categories), excluding money added to savings. Thus, rather than examining total expenditure amounts, we evaluate households who allocate similar portions of their non-saving spending to each summary expenditure category. This is an important distinction: expenditure amounts capture a household’s decisions about how much money to spend in each category and are likely to be highly influenced by hard budget constraints such as income. However, proportional expenditures reveals how households allocate their budgets across categories and provides insight into how household financial profiles differ by income (e.g., two households may have different incomes but allocate similar portions of their budgets to food, housing, and apparel).

This approach involves trade-offs when considering expenditures that are unlikely to be related to the size of the total household budget. For instance, the total amount a household spends on tobacco may be relatively fixed—two households of different income levels that smoke a pack of cigarettes per day spend the same amount but allocate different portions of their total budgets to tobacco. Despite these trade-offs, we are more interested in characterizing household budgets holistically. From our perspective, the relationship between an expenditure profile and saving is not a matter of budget size, but of how a household allocates that budget to reflect a combination of choices, tastes, and structural constraints. We are also interested in how this allocation affects savings. The hypothetical smoking households mentioned above are likely to save very differently, perhaps because smoking expenditures crowd out funds reserved for saving when they comprise a larger portion of the total budget. We attempt to isolate effects for budget allocations from hard budget constraints by controlling for household income.

## Methods

### Identifying Lifestyles

To identify lifestyles—or household financial types—from the data without a priori categorization, we use hierarchical cluster analysis, which groups households with similar spending profiles across our summary expenditure categories. We center each summary expenditure variable and then perform hierarchical agglomerative clustering using Ward’s minimum-variance method (Ward 1963), a robust general cluster analysis technique (Ferreira 2009). There are many ways to determine the number of clusters that best represents the social space, and each method requires some analytic judgment. Following general practice (Aldenderfer and Blashfield 1984; Kalleberg and Moody 1997; Moody 2001), we use the cubic cluster criterion (CCC) and compare the qualitative cluster profiles defined at each branch of the clustering solution to identify the number of clusters. The optimal value based on the CCC was nine clusters; however, because this resulted in a cluster with a single case that was otherwise qualitatively similar to the health-constrained cluster, we reassigned this case to the health-constrained cluster, resulting in an eight-cluster solution. Extensive preliminary analyses suggested this decision did not affect our results; preliminary analyses also showed that these types are robust to using various clustering methods.

Our clustering approach positions households in a relational field. We expect this space to be clumpy—to admit to clusters of cases that form unobserved groups and that act similarly because they are faced with similar resources and constraints and have internalized similar ways of acting. Households cluster into lifestyles because members have expenditure profiles that are more similar to each other than to non-cluster members. Although this approach does not explicitly incorporate other household traits such as structural position, race, family structure, or life stage, these elements are implicitly (and variably) attached to clusters as they are expressed through expenditure choices, preferences, and constraints. To parse the effects of common experiences based on attributes such as race, class, and household composition, we provide two models for each savings outcome: one with consumption clusters alone and a second holding constant household attributes. This ensures that we do not conflate attribute effects with spending profile effects and allows us to capture what is unique about the clusters beyond demographics. Crucially, we opted not to include demographic attributes in our cluster definition because lifestyles are intended to be emergent and fluid rather than ossified social categories. Households could change lifestyle over the life course, and the salience of each lifestyle may shift over historical time.

To explore the robustness of our results to large, one-time financial outlays, we conducted alternative analyses that examined annualized expenditures rather than quarterly expenditures. The cluster solution, expenditure profiles, and regression results in these alternative analyses were substantively similar to our final results. We present analyses of quarterly rather than annual expenditures to preserve the sample size and statistical power. Because of the CES rotating panel design, using annualized expenditures would have the added drawback of reducing the sample to less than 20 percent because we would exclude households with fewer than four observed quarters.

### Lifestyles and Saving

After identifying lifestyles, we explore variations across groups in saving behavior by modeling three dependent variables that together capture household saving. First, we model the logged value of current savings accounts to capture short-term saving, including the total balance of all savings accounts in banks, savings and loans, and credit unions. We do not have information on funds set aside each month as savings or investments; arguably, however, accumulated savings in short-term financial instruments better reflect true saving. Very short-term saving—saving that does not accumulate, but is spent almost immediately—provides little financial cushion of the sort that accumulates into wealth and provides long-term stability.

Second, we model the logged amount placed in individual retirement accounts (IRAs, including Keogh and SEP or self-employed IRAs) during the past 12 months to capture one type of saving for long-term plans. IRA contributions are important because they are widely available to middle-class households and require active saving from current income, an indicator that the household is deliberately setting aside financial resources for future use. Third, we model a broader measure of retirement savings that incorporates both active saving through individual retirement account contributions and more passive saving through payroll deductions. This includes the total annualized amount placed in retirement savings from individual retirement accounts, private pension pay deductions, government retirement pay deductions, and railroad retirement pay deductions. We focus on both IRAs and all retirement accounts to independently assess effects for active retirement savings, as with IRA contributions, and all retirement savings.

Our key independent variables are the financial types identified in the cluster analysis. We also control for a number of other traits well known to affect saving, including the reference person’s age and highest education level. We include a measure of logged family income that includes total before-tax income for all household members 14 years of age or older from all sources (e.g., wages, salaries, social security, unemployment compensation, workmen’s compensation, public assistance). We include nine dummy variables for income decile to ensure we are not simply identifying income differences (Slesnick 2001). We include a dummy variable indicating whether the reference person is currently enrolled in college. The reference person’s race is measured with a series of dummy variables indicating the race/ethnicity of the household head, including a multiracial category. Multiracial individuals account for a very small proportion of respondents, and experimenting with more detailed race/ethnic groups for multiracial households did not change our results. Family type is a series of dummy variables indicating marital status and number of children. The number of earners in the household captures the number of household members who are employed for pay. We control for the reference person’s hours worked and the spouse’s hours worked as well as the reference person’s employment status and sector, because these affect resource availability and access to saving instruments such as 401(k) accounts. We also control whether the household lives in an urban area, the population size of the municipality, and region of residence.

Table 1 includes descriptive statistics. Because the first dependent variable, logged savings, has a smaller sample than the other dependent variables, we include separate estimates for this subsample. Nearly all of these missing cases are due to item nonresponses, refusals, and “don’t know” responses; lower sample sizes are spread evenly across clusters, and no cluster loses an inordinate number of respondents. We report mean values for each variable except where noted; we report respondent demographics for individual traits such as education. The CES sample is representative of U.S. households on the variables we use. We use ordinary least squares (OLS) regression to model each of our dependent variables. Diagnostics revealed only marginal heteroscedasticity, but to be conservative, we present heteroscedastic-consistent standard errors. We experimented with using alternative model specifications including various maximum likelihood models, such as generalized least squares (GLS) and variants of GLS. These alternative model specifications did not change the results; we report OLS results to simplify interpretation.

## Contemporary Lifestyles

**Figure 1:**
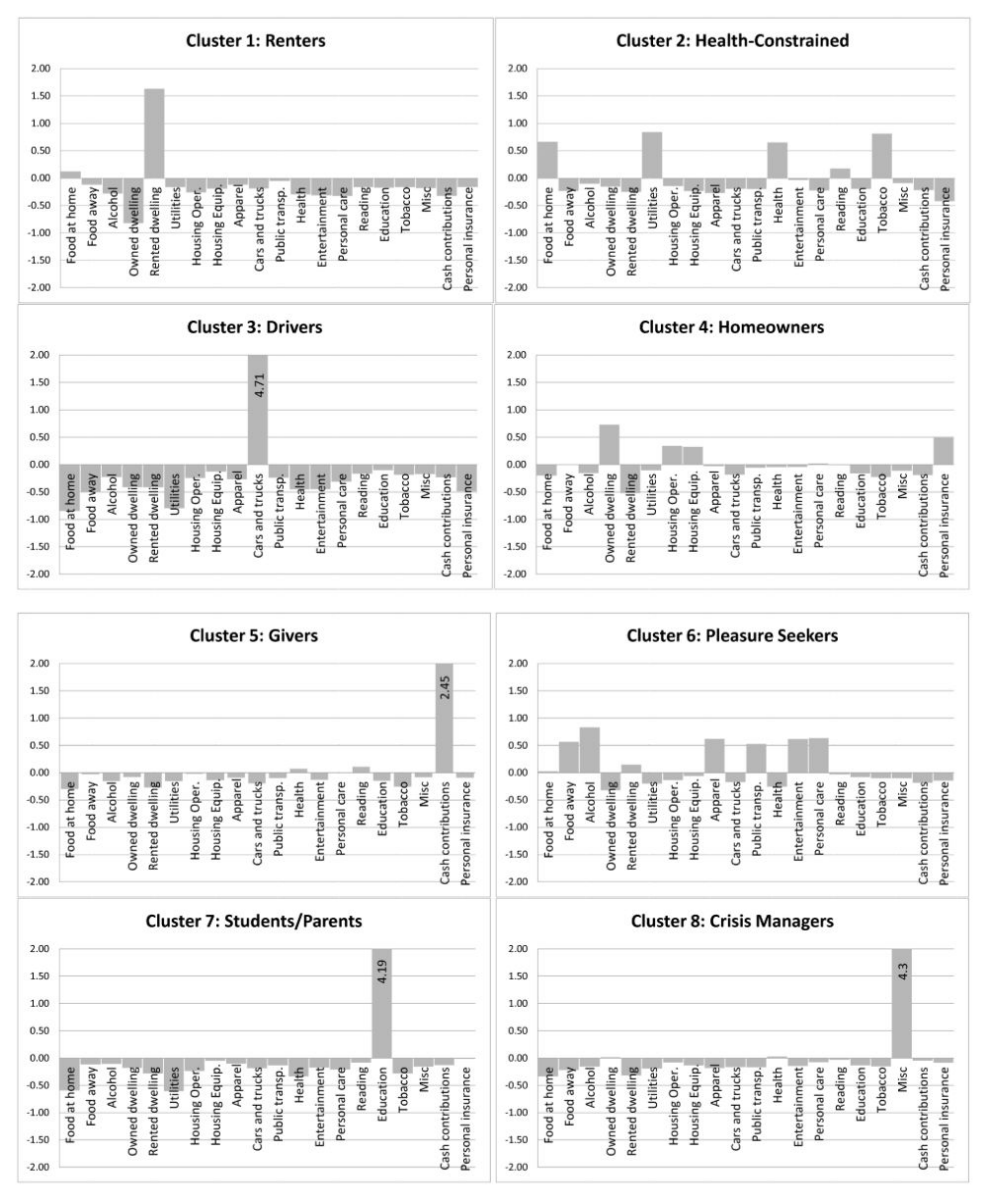
Mean standardized expenditure proportions by cluster.

Eight distinct lifestyles emerge from our data; Figure 1 provides a graphical summary of each cluster’s spending pattern. We have also included three Appendices in the supplement that offer additional detail about the clusters: Appendix A provides mean standardized expenditure proportions by cluster; Appendix B gives the clustering dendrogram; and Appendix C provides a non-standardized parallel coordinates plot based on a random sample of cases and their cluster memberships. Figure 1 shows the proportion of total expenditures accounted for by each spending type (e.g., food at home, food away from home, rent) standardized to a mean of 0 and a standard deviation of 1 and makes clear how the clusters differ from each other. We name clusters based on the dominant feature of their spending profile, though there is internal heterogeneity with respect to the behavior. For example, we refer to the first group as renters because members of this cluster, on average, spend disproportionately on their rented dwelling.^1^ Although some members in this cluster do not rent, all households in the cluster are more similar to each other than to other respondents indicating that they occupy a similar position in social space. As such, they face many of the same pressures and have access to the same resources and cultural tools as other households in this cluster.

The lifestyles identified represent positions within a multidimensional social field defined by the expenditure profiles. Table 2 includes summary statistics that underscore the sort of heterogeneity that the case-based approach identifies. Households in each cluster vary substantially on the demographic traits that social scientists typically use to model savings. That is, although spending reflects important elements of structural position, class status, geographic location, and household status (in large part because race and ethnicity, education, income, and related traits can affect both the availability and cost of goods and services), identifying groups solely based on these demographic traits is not equivalent to the lifestyles we uncover here. The within-cluster variability is also clear in a multinomial logistic regression model that predicts financial type as a function of all other attribute variables; this model only accurately classifies 40 percent of households (not shown, but available upon request). Thus, it might be tempting to imagine renters as young urban individuals who have not yet aged into homeownership, and to some extent that is true. However, Table 2 demonstrates that there is considerable variation on this theme, which is relevant to explaining household savings behavior.

**Figure 2:**
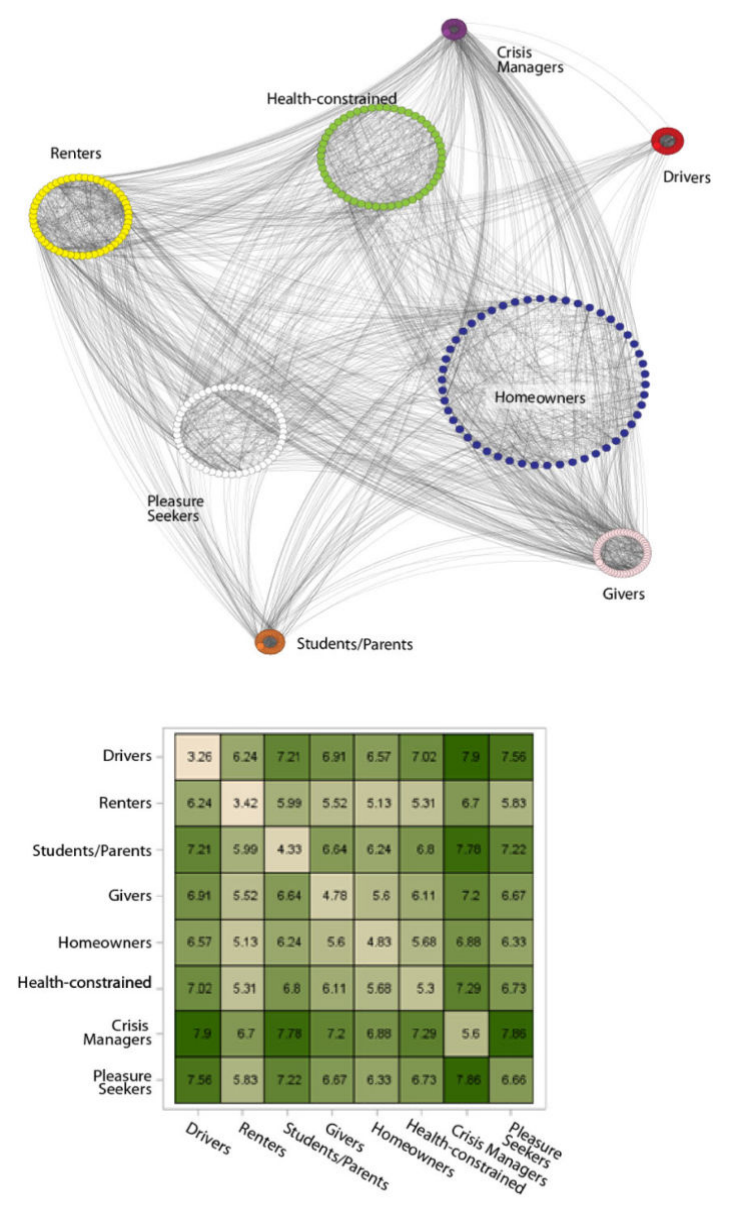
Social similarity layout for spending positions. Lines link most similar alters, circle size proportional to alter size, positioned at the cluster centroid, determined by a 2D network-based projection of the similarity space.

Figure 2 provides two representations of the field represented by the lifestyles. Whereas the dendrogram in Appendix B of the supplement represents similarity as defined by the clustering algorithm, Figure 2 uses a random sample of cases and their cluster memberships to define similarity in (a) network terms representing proximity in two-dimensional space and (b) as an aggregate similarity matrix ordered by within-group similarity. Similarity is Mahalanobis distance across the 20 expenditure categories with links drawn between closest pairs. We determined layout using a two-dimensional force-directed layout algorithm and placed nodes in circles centered on the centroid of each group to capture the relative group sizes. Admittedly, this two-dimensional layout is an imperfect fit to the 20-dimensional space on which the clustering is based, but it provides a useful representation of the social space nonetheless.

We call the first household type renters (15 percent of the sample) because they spend disproportionately on their rented dwellings. On average, members of the majority-renters group have low incomes and are headed by relatively young individuals who are likely to be in college. Yet, members of the renters group are also disproportionately black or Hispanic and are more likely than the full sample to be single parents, consistent with research finding that African Americans and Hispanics—particularly single parents—disproportionately live in economically vulnerable conditions (Shapiro et al. 2008). When these traits are grouped, there appear to be three household types that might ordinarily be considered as distinct, unrelated types: relatively young people who are still in school; low-income black and Hispanic single parents; and people living in expensive urban areas, particularly in the western United States. Of course, these subgroups overlap (e.g., some African American single parents are also students), but the tendency in most research on saving is to consider these household types separately even though their financial profiles suggest that they are similarly situated in social space.

The second lifestyle or financial type that emerges can best be described as health-constrained. Figure 1 shows that this group spends disproportionately on health and healthcare; notably, members of this group also spend disproportionately on tobacco-related products. As Table 2 shows, this is a relatively large group with more than 1,600 households (19 percent), and members tend to have low incomes. Unlike those in the renters group, the health-constrained are older than the average CES respondent and have relatively low educational levels. Members are likely to be headed by unmarried, unemployed individuals (not single parents). They live in smaller, rural cities, on average, and southerners are overrepresented. Notably, there are no clear racial or ethnic differences between those in the health-constrained group and the full sample. Because there are some spending similarities between our renters and health-constrained groups, they are socially proximate, as the dendrogram in Appendix B of the supplement and Figure 2 illustrate; thus, there may be similarities between these groups in their saving behavior as well.

We refer to the third financial type as drivers. This group (3.7 percent) includes households for whom vehicles, vehicle maintenance, and related expenses are disproportionately high, as Figure 1 shows. Members of this group have relatively high incomes and are somewhat younger than those of the average household; their race/ethnicity, educational levels, and other traits are otherwise unremarkable. One important exception is that this group tends to be married with children living in midsize cities and towns. One interpretation is that this group includes soccer moms and dads, that is, suburban, middle-class parents who spend considerable amounts of time transporting their young children to sporting and other activities. Consistent with this interpretation, households in this group are also more likely to have college-educated heads and two earners in the household. Americans are driving more than in previous decades, but a good deal of this is accounted for by driving by choice, particularly driving to take children to school, practice for sporting events, and other extracurricular activities (Handy, Weston, and Mokhtarian 2005). Our drivers category captures the financial implications of this reality. There is also evidence that parental spending on children has increased considerably since the 1970s, but spending varies across households (Kornich and Furstenberg 2013). Our drivers group reflects some of this cross-household difference that would be masked by variable-centered approaches.

We call the fourth lifestyle that emerged from our cluster analyses homeowners. Figure 1 illustrates that costs associated with the primary (owned) residence are significant expenditures for this group; these costs include the dwelling itself, related housing expenses (e.g., cleaning supplies, landscaping, yard supplies), furniture, household textiles (e.g., linens, floor coverings), major appliances, and housing equipment (e.g., lamps, power tools, telephones, home computers). This group also spends disproportionately on personal insurance, including homeowners insurance and personal liability insurance policies, which are often held to cover liability claims associated with real estate. Table 2 illustrates that the homeowners group is the largest lifestyle group with 2,712 households (31 percent). Because of its size, the homeowners group influences the mean values shown in Table 2 more than other groups. Members of these households tend to be highly educated, white, married, and to have two earners. The total household income for this group is not dramatically higher than average, suggesting that this is not simply a high-socioeconomic status group. Rather, married-couple households across the income spectrum are well represented in this group.

We refer to the fifth group as givers (8 percent) because gifts to charitable organizations, particularly religious organizations, are the single most significant expense for these households. Nearly 90 percent of U.S. households report donating some amount of money to a charitable organization each year, but research has documented important demographic differences in donating (Havens, O’Herlihy, and Schervish 2006). Consistent with patterns in the research, Table 2 shows that households in the givers cluster tend to be married with one working spouse, have high educational levels, and have moderately high incomes. Interestingly, the emphasis on giving to religious organizations and the demographic traits of this group suggest that households in this cluster may be conservative Protestants (CP) or Mormons (Latter Day Saints or LDS). CPs and LDS tend to give generously to religious causes (Keister and Sherkat 2013) and tend to have traditional gender divisions of labor, with husbands working and wives either not in the labor force or working part-time (Lehrer 1995, 1996). Although religious groups have been studied together, our givers group is broader and incorporates households that would not typically be included together in variable-centered approaches.

The sixth and seventh groups both contain large numbers of young households, but the expenditure profiles of these households produce two distinct lifestyle groupings: the sixth group is perhaps best described as pleasure seekers (17 percent). These respondents spend relatively similar amounts of money on a related set of goods and services, including dining away from home, alcohol, clothing, public transportation, entertainment, and personal care. Members of this group are younger than the average household in this sample and are more likely to be single. More than one half of this group is employed in private companies, and 40 percent live in large cities (with populations exceeding 4 million).

The seventh group also includes large numbers of young, urban households, but is quite distinct from the sixth group. Group seven is best described as students and their parents and contains households that spend disproportionately on education and education-related goods and services. As the name implies, this small (3 percent) group includes many young adults who are still in school as well as their parents who are helping to pay for education-related expenses. This category also likely includes adults who are still enrolled (or enrolled again) in colleges and universities, consistent with a growth in the number of nontraditional adults who are pursuing higher education. Because these very different household types are included in this cluster, the demographic traits tend to be bimodal; for example, the group includes young and middle-aged adults, those with lower and higher incomes, and single and married households. The dendrogram in Appendix B of the supplement suggests groups six and seven occupy unique positions in the social space as well; pleasure seekers are located closer to renters and the health-constrained, and parents and students are positioned more closely to givers and drivers.

Our eighth and final group is also small (2.7 percent) and perhaps best called crisis managers, spending disproportionately on an eclectic set of expenses labeled “miscellaneous” by the CES (see Figure 1). This spending category includes finance charges at banks and related financial organizations (23 percent of total expenses in this category), legal fees excluding those associated with real estate purchases (22 percent of the total), property expenses for investment properties (17 percent of the total), funeral and burial expenses (11 percent of the total), and other miscellaneous expenses (27 percent of the total).^2^ We acknowledge that the diverse nature of these expenses makes it difficult to categorize households in this group, but we opted to retain the group because the spending patterns of these households have high within-group similarity and are highly distinct from other households. Our decision to refer to these households as crisis managers reflects the fact that crisis-related expenses account for more than one half of expenses in this category: bank fees, legal fees, and funeral expenses account for 56 percent of total miscellaneous expenses. Paying bank fees suggests that a household is experiencing either temporary or ongoing financial duress. Legal fees can also signal a crisis (e.g., criminal trouble, divorce, child custody), although these fees may also be routine (e.g., estate planning). Funeral expenses are clearly a sign of crisis, albeit a crisis that has, in part, ended. Table 2 suggests that these households have relatively high incomes, are older, and are somewhat more highly educated than the typical CES respondent; otherwise, households from a large range of demographic groups and regions of the country are represented in this cluster. One trait worth noting is that these households have average rates of self-employment; that is, business-related expenses or expenses associated with business start-up are not responsible for the spending profiles of these households, providing additional evidence that crisis spending may be the underlying factor that relates these households.

## Findings: Lifestyles and Saving

The descriptive statistics in Table 2 suggest that there are important differences in household saving across the financial types: homeowners, givers, and students/parents have particularly high savings, whereas renters and the health-constrained have low savings. To explore whether these patterns extend beyond what would be expected given their demographic composition, we conducted multivariate analyses using the eight clusters as independent variables. Most of the effects from lifestyles are robust after control variables are included, suggesting that our approach to expenditure profiles reveals unique information about savings that would be lost when demographic and employment characteristics alone are used. The controls moderate the strong-program claims that treat all elements as features of the social space characterizing positions; by contrast, we ask only whether it is possible to identify a unique association—a way of living—beyond the well-established demographic and employment associations. Table 3 contains results of OLS models of our three saving measures: the logged value of current savings accounts; the logged amount contributed to IRAs during the past 12 months; and the annualized amount contributed to all retirement accounts, including IRAs and pay deductions for government pensions, private pensions, and railroad retirement. The first two models use savings accounts as the dependent variable, and model 1 includes only our main test variables, the indicators of the expenditure clusters. We use the majority homeowners group as our omitted category because this is the largest cluster and has clearly higher savings and retirement account values compared with households in the other clusters. Using other clusters as the omitted group did not change the result substantively.

The first multivariate model (Table 3, model 1) shows that households in five of the clusters all save significantly less than homeowners: renters save the least, followed by the health-constrained, pleasure seekers, crisis managers, and drivers. Model 2 shows that all but one of these associations hold when we control for individual and household demographic traits, family structure, income, employment status, and characteristics of the place of residence. The strength of the coefficients for the financial types is reduced and significantly different from the base model when we add the controls, but this is not surprising given that the control variables are all associated with saving in ways that are consistent with well-established patterns in the literature and correlated with financial types. The relatively high *R*^2^ for model 2 suggests that our model captures significant amounts of the variation in this dependent variable. Notably, we find no significant difference between the short-term saving of givers or students/parents and homeowners; we also find that the effect of being a driver does not hold when the controls are included. This pattern is consistent with the evidence from the cluster analysis that givers, students/parents, and drivers are all located closer to homeowners in the social space pictured in Appendix B of the supplement, whereas renters, the health-constrained, and pleasure seekers occupy different spaces. Some effect sizes are relatively large; as Figures 1 and 2 demonstrate, there is considerable social distance among the clusters, and the large effect sizes likely reflect this distance. Moreover, exponentiating the coefficients (to get expected percent difference between the category and the homeowner reference group) indicates that some groups save quite a bit more than others; however, these differences are not out of range from the coefficients for other variables such as race and family structure. More importantly, the variation across groups is consistent with the wealth literature that shows that some groups really do save considerably more than others.

There are slightly different associations between lifestyle and long-term IRA saving. Model 3 shows that compared to homeowners, renters have the lowest IRA savings, followed by health-constrained, crisis managers, givers, and pleasure seekers. Model 4 introduces the control variables and shows that the associations evident in model 3 hold with the exception of pleasure seekers: the coefficient for this cluster becomes insignificant. Another important difference between our models of savings accounts (models 1 and 2) and our models of IRA savings (models 3 and 4) is that givers save less for retirement than homeowners, but they save comparable amounts in short-term savings or savings accounts, suggesting that charitable contributions affect short- and long-term saving differently. Also noteworthy is that students/parents and drivers save amounts that are comparable to those of homeowners. To assess whether sample bias affects our results, we estimated the long-term savings models for the constrained sample used in models 1 and 2 for short-term savings accounts; results were substantively identical to the full-sample models.

Finally, models 5 and 6 estimate associations between lifestyle and total retirement (long-term) savings. Model 5, the base model, shows that compared with the homeowners category, the remaining seven lifestyle clusters have lower annualized total retirement saving contributions. We again find that the health-constrained and renters save the least, followed by pleasure seekers, givers, crisis managers, and drivers. Adding covariates removes statistical significance for one category—crisis managers—but the effects for all other clusters remain. Substantively, renters, the health-constrained, and pleasure seekers save the least for long-term total retirement. This effect mirrors some of the lifestyle clusters’ negative short-term savings from model 2. The covariates for education, income, and race are also in the expected direction. In addition, government employees save more for retirement, whereas the self-employed save less—effects were not significant in models 3 and 4 capturing active retirement savings through IRAs. This effect is likely due to the predominance of payroll deduction-based pensions, which are absent for many who are self-employed.

In supplementary analyses (results available on request), we compared our cluster-based regression models to traditional variable-centric models, regressing each of our dependent variables on the individual expenditure categories used to construct our clusters. Results support our interpretation that a household-centered approach to lifestyles reveals unique information that would remain unidentified under traditional variable-based approaches. First, clusters that heavily reflect single expenditure categories (drivers, parents and students, and renters) do not act as dummy variables for those expenditure categories. In each of these cases, the expenditure categories alone are not associated with savings. However, when we consider these in the context of other expenditures as part of a multidimensional lifestyle, each is negatively associated with at least one type of savings. Second, several individual expenditure categories are associated with savings in the opposite direction from what is revealed when considering them as part of a clustered lifestyle. For example, expenditures on alcohol, food away from home, entertainment, apparel, and personal care are each positively associated with at least one form of savings. Yet, our cluster analysis identifies a group of households that exhibits high proportional expenditures in each of these categories, and we find that inhabiting this position in the relational space is negatively associated with short-term and long-term savings (models 2, 6). That is, when the marginal effects of these expenditures are considered in isolation, they are correlated with increased savings. However, our analysis recognizes that a handful of households that spend disproportionately on one of these categories also spend disproportionately on the other categories; we reduce this multidimensionality into identifiable lifestyle clusters. These clusters have implications for savings that are quite distinct from any one expenditure category alone. This further supports our argument that a focus on lifestyles as a position in the expenditure behavior space reveals unique insights that would otherwise be overlooked.

Considering these lifestyle clusters as a household’s position in an expenditure behavior space offers some insights into potential mechanisms underlying the differences observed in the regression models. Close inspection of the dendrogram (Appendix B of the supplement) reveals the relative proximity in the expenditure space roughly aligns with savings rate order. Renters, the health-constrained, and pleasure seekers are relatively proximate in the expenditure space, and they are more distant from homeowners, crisis managers, givers, and students/parents. Notably, this mirrors the substantive findings. Renters, the health-constrained, and pleasure seekers have significant and sizably less short-term savings in cash accounts (model 2) as well as long-term retirement savings (model 6) than do homeowners. Similarly, givers and students/parents do not differ from homeowners in their short-term savings, and although the effects are statistically significant for long-term savings, they are substantively smaller than the more distant clusters.

The relationship between our income control variable and savings—across models—is particularly important. It is commonly assumed that income and accumulated savings are more highly correlated than they are. In fact, the correlation between income and savings is relatively low, as we mentioned in the introduction. Even the correlation between capital gains income and total net worth is only .35 (Keister 2014). This highlights the fact that some people with remarkably low incomes still manage to save for the future while many with very high incomes save very little. It is precisely this within-income variability in savings that makes the lifestyles we identify so substantively interesting. We are not capturing just another indicator of income; rather, these clusters capture a way of approaching money that carries over to how they save, even for households with the same incomes. Our spending categories are also based on proportions of the respondent’s total non-savings expenditures. Thus, it is possible for two households to have the same clustering profile (with a high proportion of their spending on, say, rent) but to save very different amounts. Moreover, our dependent variables are not merely the difference between income and expenditure; rather, they indicate the allocation of accumulation of savings over different timespans. Thus, even two households that save the same amount might allocate funds differently across savings account, IRAs, and total retirement.

## Conclusion and Discussion

Thinking of people as cases that are proximate in a behavior space rather than as markers of single variables has a long history in sociology, but finding analytically tractable ways to implement this idea is challenging. We took seriously the notion that people can be thought of as whole packages and used household spending allocations to identify groups with similar lifestyles. We identified eight positions in the financial behavior space and named them to reflect a dominant trait of the majority members (renters, the health-constrained, drivers, homeowners, givers, pleasure seekers, students and parents, and crisis managers). We then explored how membership in one of these groups is associated with saving, an important measure of short- and long-term household well-being. We found that households in the renters and health-constrained groups save less than the homeowners across all three categories. Pleasure seekers and crisis managers also consistently save less than homeowners, although the findings for retirement savings (IRA savings for pleasure seekers and total retirement savings for crisis managers) are not significant after we control for other correlates of savings. We find that drivers, students and parents, and givers save about the same in cash accounts as homeowners do, but these three groups save less for retirement than homeowners. This research also adds to the literature on inequalities in household saving by demonstrating that, despite its many strengths, prior research might be limited by its focus on single-variable explanations of saving. Our analyses demonstrate that considering a household’s lifestyle adds to our understanding saving.

Our findings cannot speak directly to the mechanisms that relate lifestyle to saving, but the results suggest that the three mechanisms we proposed are plausible. First, budget constraints likely play a key role in our renter, health-constrained, and crisis managers clusters, who all have lower saving even after income, education, and employment status are controlled. In all cases, these households spend disproportionately on features that indicate a lack of flexibility—such as healthcare and rent—or occupy a generally precarious financial position (characterized by high legal and finance fees), suggesting lifestyles lived at the financial margins. Given these basic constraints, saving any amount of money is challenging. High spending on educational costs, characteristic of our student/parents group, is also a clear budget constraint, but seems to only account for deficits in long-term total retirement savings. This may indicate a general knowledge and access effect; that is, these households may postpone long-term savings for current investment in education. Similarly, drivers allocate a large fixed portion of their budget to travel and commuting expenses, but this seems to have strong negative effects only for the longest term retirement savings, perhaps reflecting a direct tradeoff between current expenses and saving.

Second, a knowledge- and access-based mechanism is primarily practical knowledge—pragmatic insights about best practices and opportunities—rather than simple human capital (which is directly controlled for in our models). Understanding even basic savings and checking accounts can be complicated for those with limited education or experience dealing with formal financial organizations; IRAs and related accounts can be challenging even for the most educated, financially savvy consumers. Add to this the tax implications and rules regarding withdrawals and associated penalties of saving in these accounts, and it is not surprising that many Americans shy away from them altogether. Homeowners have the highest long-term savings rates and, by virtue of having navigated the home-buying process, likely have greater direct experience with financial institutions as well as informal networks of neighbors who have all interacted directly with significant financial institutions. Similarly students/parents are actively engaged in human capital investment and are more likely to interact with other educated friends and colleagues, increasing potential access to financial knowledge networks. In contrast, crisis managers’ experience with financial institutions appears to be negative, at least so far as it is reflected in extensive fees and service charges.

Finally, saving is an important marker of delayed gratification: saving requires postponing current consumption to enable future consumption. Although this approach to money reflects financial knowledge, it is also characteristic of certain lifestyles. For example, the pleasure-seeker group consists of people who spend disproportionately on eating out, entertainment, alcohol, and apparel; this likely reflects a sense that the future will take care of itself. Evidence suggests smokers are more likely than nonsmokers to discount future rewards and that, as a result, educational level and other outcomes that indicate postponed consumption are inversely associated with discounting in smokers (Jaronia et al. 2004). Although our health-constrained cluster is more than an indicator of smoking, tobacco purchases do account for an important part of these households’ expenditures; thus, discounting may help explain the association we find between being in the health-constrained cluster and saving. On the other hand, student/parents are investing directly in the future and thus have traded current long-term saving for future earning while still maintaining generally high short-term saving levels. Crisis managers are situationally unable to delay consumption as they likely occupy a position characterized by a short-term necessary spending servicing past debts and immediate crises. Givers are interesting: our results suggest that they are donating funds now at the cost of long-term saving. Other work shows that, at least for conservative Protestants, this is a conscious approach to savings with the expectation that future problems are in God’s hands (Keister, 2011).

Of course, it is important to note that there may not be a single social process corresponding to occupancy in a region of the social space that explains the saving differential across our observed financial clusters or can be distilled into a single variable. In fact, the strong proponents of case-based approaches tend to avoid such speculation entirely as social practices are seen to emerge historically from a long pattern of earlier actions that reinforce position in the space. For example, reliance on spot transactions, frequent job-change, or residential instability might lead to a distrust of financial institutions entirely, which is then passed to children as part of the taken-for-granted fabric of their lives. This feature might manifest for those in our majority-renters cluster even if they have the financial reserves necessary to purchase a home or otherwise invest. Unfortunately, we cannot explore such possibilities directly with available data. Future work, particularly good financial ethnography, would be better suited to identifying such features. That is, we acknowledge that our treatment of the relationship between lifestyles and saving stops short of completely identifying the mechanisms at work here and both the between- and within-cluster heterogeneity in saving; future research might usefully start with these two points and explore these issues in more depth.

There are other directions in which this work could also be extended. First, future research might examine whether households move among lifestyles over their lives. If this is the case, the meaning of our lifestyles might diverge from Bourdieu’s conception on which we rely. The age grading in our results suggest that there is movement among some of our categories, but we are unable to study this issue because the CES is cross-sectional and other currently available spending data is less complete than the CES. Future research might also consider applying clustering approaches to other explanatory variables (e.g., education, occupation) to allow analysts to explore whether this strategy offers a more parsimonious way to understand household behavior. Explicit comparisons between the variable-and case-centered results in studies of multiple outcomes would help clarify the advantages and weaknesses of each strategy. We studied lifestyles and spending, a fixed-sum outcome for households, but other work would usefully examine how lifestyles relate to various types of outcomes. Consistent with this, varying how lifestyles are defined would be useful both theoretically and methodologically. Using spending to define ways of living is useful because it closely mirrors cultural taste and identity and is closely tied to a particular domain of social life (Savage and Gayo 2011). Expanding on this definition by incorporating other elements of demographical position simultaneously would allow researchers to better understand the complex relationship between case- and variable-centered approaches.

## Figures and Tables

**Table 1:** Descriptive Statistics for Independent and Dependent Variables.

	Mean (full sample)	Std. Dev. (full sample)	Mean (subsample)	Std. Dev. (subsample)
**Dependent Variables (2011 $)**				
Ln savings (n = 5,657)			4.27^a^	4.32
Total savings amount			13, 092.22	50, 688.42
Ln IRA (n = 8,536)	0.55	2.07		
Amount placed in retirement account in last 12 months	546.01	4,145.19		
Ln total retirement (n = 8,536)	1.32	3.05		
Amount placed in all retirement accounts annualized	1,174.73	5,138.22		
**Independent Variables**				
Total HH income (2011 $)	53,060^a^	68,727	57,006^a^	70,432
Age	49.86	17.47	48.37	17.26
Education				
Less than high school	0.14	0.34	0.15	0.36
High school	0.25	0.43	0.24	0.42
Some college	0.21	0.41	0.21	0.41
College	0.29	0.45	0.28	0.45
Advanced degree	0.11	0.32	0.10	0.31
In college	0.09	0.29	0.10	0.30
Race				
White	0.68	0.47	0.67	0.47
Black	0.12	0.33	0.12	0.33
Hispanic	0.13	0.34	0.15	0.35
Native American	0.01	0.06	0.00	0.06
Asian or Pacific Islander	0.05	0.23	0.05	0.22
Multiracial	0.01	0.10	0.01	0.10
Family type				
Husband and wife only	0.20	0.40	0.18	0.38
Husband and wife with children <17	0.16	0.37	0.17	0.37
Husband and wife with children >17	0.07	0.26	0.07	0.25
Other husband and wife	0.05	0.22	0.05	0.21
Single parent	0.06	0.23	0.07	0.25
Single person	0.30	0.46	0.31	0.46
Other family type	0.15	0.36	0.16	0.36
Number of earners	1.24	0.94	1.23	0.93
Hours worked	26.13	21.35	26.29	21.33
Spouse’s hours worked	13.63	20.47	13.18	20.41
Employment				
Not employed	0.34	0.48	0.34	0.47
Private company	0.48	0.50	0.49	0.50
Government	0.12	0.32	0.12	0.33
Self-employed	0.06	0.24	0.06	0.23
Self-employed without pay	0.00	0.02	0.00	0.03
Urban	0.95	0.22	0.95	0.22
Population size				
Greater than 4 million	0.36	0.48	0.35	0.48
1.2–4 million	0.24	0.42	0.24	0.42
.33–1.19 million	0.06	0.25	0.06	0.23
125,000–329,900	0.22	0.41	0.23	0.42
Less than 125,000	0.12	0.32	0.12	0.33
Region				
Northeast	0.19	0.39	0.19	0.39
Midwest	0.23	0.42	0.22	0.42
South	0.35	0.48	0.34	0.47
West	0.23	0.42	0.24	0.43

a Median savings is $3.93; median income for the full sample is $34,000, and median income for the subsample is $38,000.

**Table 2:** Summary Statistics by Cluster.

Group Name	Total Sample	Cl 1 Renters	Cl 2 Heath- Constrained	Cl 3 Drivers	Cl 4 Home- owners	Cl 5 Givers	Cl 6 Pleasure Seekers	Cl 7 Students/ Parents	Cl 8 Crisis Managers
**Dependent Variables**									
Ln savings (n = 5,657)	4.27 (4.32)	2.37* (3.51)	2.89* (3.89)	4.73 (4.25)	5.66* (4.32)	5.64* (4.50)	4.06 (4.26)	5.84* (4.23)	4.75 (4.21)
Ln IRA (n = 8,536)	0.55 (2.07)	0.19* (1.17)	0.23* (3.89)	0.69 (2.22)	0.90* (2.64)	0.50 (2.00)	0.54 (2.00)	0.80* (2.53)	0.43 (1.74)
Ln tot. retirement (n = 8,536)	1.32 (3.06)	0.59* (2.06)	0.52* (1.94)	1.61 (3.28)	2.16* (3.74)	1.26 (3.01)	1.12* (2.80)	1.66* (3.46)	1.54 (3.23)
**Independent Variables**									
Income	53,060 (68,727)	30,851* (34,546)	29,189* (32,654)	65,644* (65,903)	72,557* (83,296)	63,955* (86,645)	49,250* (64,530)	72,819* (82,577)	62,509* (68,109)
Age	49.86 (17.47)	45.05* (17.80)	56.53* (17.66)	45.12* (16.04)	50.03 (15.28)	55.52* (17.44)	45.87* (18.31)	41.23* (14.86)	53.38* (16.25)
Education									
No H.S. diploma	0.14	0.20*	0.22*	0.11	0.08*	0.10*	0.14	0.04*	0.09*
High school	0.25	0.28*	0.35*	0.23	0.21*	0.25	0.23*	0.10*	0.25
Some college	0.21	0.23	0.20	0.23	0.19*	0.18	0.22	0.34*	0.22
College	0.29	0.23*	0.18*	0.33	0.36*	0.31	0.30	0.38*	0.29
Advanced degree	0.11	0.06*	0.04*	0.09	0.16*	0.16*	0.11	0.15	0.15
In college	0.09	0.11	0.05*	0.10	0.07	0.07	0.14*	0.35*	0.06
Race									
White	0.68	0.49*	0.71*	0.70	0.73*	0.72*	0.66	0.76*	0.73
Black	0.12	0.19*	0.14*	0.09	0.09*	0.14	0.13	0.05*	0.10
Hispanic	0.13	0.23*	0.11*	0.14	0.11*	0.09*	0.15	0.07*	0.08*
Native American	0.00	0.01	0.00	0.00	0.00*	0.00	0.01*	0.00	0.00
Asian	0.05	0.08*	0.03*	0.04	0.06	0.04	0.05	0.10*	0.07
Multiracial	0.01	0.01	0.01	0.02	0.01	0.01	0.01	0.01	0.01
Family type									
Husband & wife	0.20	0.10*	0.22	0.18	0.24*	0.30*	0.18*	0.17	0.21
Husband & wife w/children <17	0.16	0.13*	0.07*	0.25*v*	0.25*	0.15	0.12*v*	0.16	0.14
Husband & wife w/children >17	0.07	0.04*	0.06*	0.11*	0.10*	0.05*	0.05*	0.19*	0.06
Other Husband & wife	0.05	0.04	0.06*	0.06	0.06*	0.04	0.03*	0.06	0.07
Single parent	0.06	0.09*	0.06	0.05	0.05*	0.03*	0.06	0.03*	0.06
Single person	0.30	0.40*	0.35*	0.18*	0.19*	0.33	0.41*	0.29	0.33
Other family type	0.15	0.19*	0.19*	0.18	0.12*	0.11*	0.15	0.10*	0.14
Number of earners	1.23 (0.94)	1.17* (0.93)	0.86* (0.93)	1.52* (0.91)	1.50* (0.92)	1.10* (0.88)	1.14* (0.85)	1.64* (0.97)	1.17 (0.88)
Hours worked	26.12 (31.35)	25.28 (20.22)	16.64* (20.64)	30.13* (20.23)	30.72* (20.29)	24.49* (22.61)	27.71 (21.32)	32.01* (19.43)	25.16 (22.19)
Spouse’s hours worked	13.63 (20.46)	8.80* (17.08)	8.24* (17.33)	18.01* (22.21)	19.55* (21.99)	13.69 (20.74)	10.16* (18.72)	20.92* (22.47)	14.03 (21.36)
Employment status									
Not employed	0.34	0.34	0.56*	0.26*	0.25*	0.40*	0.31*	0.19*	0.37
Private company	0.47	0.54*	0.34*	0.54*	0.50*	0.43*	0.52*	0.57*	0.45
Government	0.12	0.09*	0.07*	0.13	0.15*	0.10	0.12	0.14	0.12
Self-employed	0.06	0.04*	0.03*	0.06	0.09*	0.06	0.06	0.10*	0.06
Self-employed without pay	0.02	0.00	0.00	0.00	0.00	0.00	0.00	0.00	0.00
Urban	0.95	0.97*	0.91*	0.95	0.96	0.95	0.96	0.97	0.97
Population size									
Over 4 million	0.36	0.45*	0.24*	0.30*	0.39*	0.31*	0.40*	0.37	0.31
1.2–4 million	0.23	0.25	0.22	0.21	0.25	0.22	0.22	0.21	0.30*
.33–1.19 million	0.06	0.05*	0.08*	0.08	0.06	0.08	0.06	0.06	0.06
125,000–329,900	0.22	0.18*	0.26*	0.25	0.20*	0.29*	0.20*	0.22	0.23
Less than 125,000	0.16	0.06*	0.19*	0.16	0.09*	0.10	0.11	0.13	0.09
Region									
Northeast	0.19	0.18	0.18	0.17	0.20	0.17	0.22*	0.26*	0.22
Midwest	0.23	0.16*	0.23	0.26	0.23	0.24	0.24	0.30*	0.21
South	0.35	0.34	0.43*	0.37	0.33	0.37	0.29*	0.21*	0.32
West	0.23	0.32*	0.15*	0.19	0.24	0.22	0.24	0.23	0.24

n	8,536	1,274	1,611	320	2,712	677	1,419	294	229

* p<0.05; (S.D. of the mean reported in parentheses for continuous measures)

Note: for values on a given variable, the significance test compares cases within the respective expenditure cluster to cases outside the cluster. Therefore, statistically significant differences are not tested against a reference category, rather each cluster is tested against the rest of the sample. Continuous variables are tested using a t-test and nominal variables are tested using a chi-square test.

**Table 3:** Financial Types and Saving: OLS Regression Models.

	DV: Ln Savings n = 5,657^a^		DV: Ln IRA n = 8,536^b^		DV: Ln Total Retirement n = 8,536^c^	
	Model 1	Model 2	Model 3	Model 4	Model 5	Model 6
Intercept	5.65* (0.10)	1.02† (0.49)	0.89* (0.05)	0.59* (0.20)	2.16* (0.07)	0.37 (0.28)
Expenditure Cluster (ref. = cluster 4–homeowners)						
Cluster 1—Renters	3.28* (0.15)	1.05* (0.15)	0.71* (0.04)	0.20** (0.06)	1.56* (0.09)	0.56* (0.09)
Cluster 2—Health-Constrained	2.76* (0.15)	−0.88* (0.15)	0.67* (0.06)	0.15* (0.06)	1.63* (0.08)	0.52* (0.08)
Cluster 3—Drivers	0.92* (0.29)	0.09 (0.26)	0.21 (0.13)	0.08 (0.13)	0.55* (0.19)	0.41† (0.18)
Cluster 5—Givers	0.03 (0.25)	0.35 (0.21)	0.39* (0.09)	0.27* (0.09)	0.89* (0.16)	0.59* (0.13)
Cluster 6 —Pleasure Seekers	1.59* (0.17)	0.26† (0.13)	0.36* (0.07)	0.09 (0.07)	1.03* (0.10)	0.51* (0.09)
Cluster 7—Students/Parents	0.18 (0.33)	0.42 (0.31)	0.10 (0.16)	0.12 (0.15)	0.49† (0.21)	0.47† (0.19)
Cluster 8—Crisis Managers	0.93* (0.34)	0.60† (0.30)	0.47* (0.13)	0.36* (0.13)	0.61* (0.23)	0.34 (0.21)
Income deciles (ref. = decile 1)						
Decile 2		0.65* (0.24)		0.14* (0.05)		0.31* (0.06)
Decile 3		0.32 (0.23)		0.17* (0.04)		0.39* (0.06)
Decile 4		1.04* (0.23)		0.10* (0.04)		0.38* (0.05)
Decile 5		1.59* (0.24)		0.21* (0.05)		0.59* (0.07)
Decile 6		1.99* (0.25)		0.31* (0.05)		0.98* (0.09)
Decile 7		3.02* (0.25)		0.58* (0.08)		1.63* (0.11)
Decile 8		3.41* (0.26)		0.65* (0.08)		1.99* (0.13)
Decile 9		4.27* (0.27)		1.22* (0.11)		2.78* (0.15)
Decile 10		5.45* (0.29)		1.49* (0.12)		3.16* (0.16)
Age		0.02* (0.00)		0.01* (0.00)		0.00 (0.00)
Education (ref. = less than high school)						
High school		0.522* (0.15)		0.05 (0.04)		0.15† (0.07)
Some college		0.99* (0.16)		0.03 (0.06)		0.00 (0.09)
College		1.42* (0.17)		0.10 (0.06)		0.14 (0.08)
Advanced degree		1.97* (0.22)		0.37* (0.11)		0.37* (0.14)
In college		0.31 (0.18)		0.04 (0.08)		0.08 (0.11)
Race (ref. = white)						
Black		1.19* (0.14)		0.14* (0.05)		0.12 (0.08)
Hispanic		1.07* (0.14)		0.22* (0.05)		0.22† (0.08)
Native American		2.22* (0.63)		0.46* (0.09)		0.54 (0.41)
Asian or Pacific Islander		0.52† (0.24)		0.04 (0.11)		0.13 (0.15)
Multiracial		1.24† (0.49)		0.09 (0.19)		0.48 (0.32)
Family Type (ref. = husband and wife with children <17)						
Husband and wife only		0.94* (0.17)		0.26* (0.09)		0.21 (0.13)
Husband and wife with children >17		0.20 (0.24)		0.06 (0.11)		0.01 (0.16)
Other husband and wife		0.52† (0.25)		0.02 (0.11)		0.16 (0.17)
Single parent		0.73* (0.22)		0.32* (0.10)		0.54* (0.15)
Single person		0.34 (0.19)		0.33* (0.09)		0.57* (0.12)
Other family type		0.35 (0.21)		0.32* (0.09)		0.41* (0.14)
Number of earners		0.17 (0.11)		0.10† (0.04)		0.14† (0.06)
Hours worked		0.01 (0.01)		0.01† (0.00)		0.02* (0.00)
Spouse’s hours worked		0.01 (0.00)		0.01* (0.00)		0.02* (0.00)
Employment status (ref. = private company)						
Not employed		0.26 (0.25)		0.10 (0.11)		0.09 (0.16)
Government		0.22 (0.15)		0.00 (0.09)		0.77* (0.13)
Self-employed		0.42 (0.23)		0.24 (0.12)		0.53* (0.14)
Self-employed without pay		0.23 (2.34)		0.43* (0.14)		0.87† (0.39)
Urban		0.15 (0.25)		0.13 (0.11)		0.25 (0.15)
Population size (ref. = Over 4 million)						
1.2–4 million		0.16 (0.13)		0.03 (0.06)		0.19† (0.08)
.33–1.19 million		0.70* (0.23)		0.02 (0.10)		0.16 (0.13)
125,000–329,009		0.09 (0.13)		0.03 (0.06)		0.21† (0.08)
Less than 125,000		0.24 (0.19)		0.12 (0.09)		0.21 (0.11)
Region (ref. = Northeast)						
Midwest		0.10 (0.16)		0.06 (0.07)		0.20† (0.09)
South		0.34† (0.14)		0.05 (0.07)		0.02 (0.09)
West		0.26 (0.14)		0.07 (0.07)		0.27* (0.09)

*R*^2^	0.09	0.33	0.02	0.10	0.05	0.23

* p<0.01,

† p<0.05 (significance computed with heteroscedastic-robust standard errors, SE in parentheses)

a Ln savings: total balance of savings and checking accounts.

b Ln IRA: total money invested in an individual retirement plan, such as an IRA or Keogh, by all household members in the past 12 months.

c Ln total retirement: sum of the amount placed in an IRA, amount of government retirement deducted from pay, amount of private pensions deducted from pay, and amount of railroad retirement deducted from pay, all annualized.

## Footnotes

1 Labeling the clusters with a single word is not ideal because it simplifies information and implies essentialist interpretations in the very way we are arguing against; however, we use the short-phrase names to avoid overly cumbersome language in the remainder of the text.

2 Finance charges are fees for using financial services; these do not include purchases of investment instruments that could also be considered saving (e.g., certificates of deposit [CDs] or savings bonds). We do not use financial instruments as dependent variables because they have large numbers of missing values, and we exclude them in constructing clusters.
